# Annual phenology and migration routes to breeding grounds in western-central North Pacific sei whales

**DOI:** 10.1038/s41598-024-61831-8

**Published:** 2024-05-16

**Authors:** Kenji Konishi, Shingo Minamikawa, Lars Kleivane, Megumi Takahashi

**Affiliations:** 1 The Institute of Cetacean Research, 4-5, Toyomi-cho, Chuo-ku, Tokyo, 104-0055 Japan; 2 Fisheries Resources Institute, Japan Fisheries Research and Education Agency, 2-12-4, Fukuura, Kanazawa-ku, Yokohama-shi, Kanagawa 236-8648 Japan; 3 LKARTS-Norway, Skutvik Landhandel, 8290 Skutvik, Norway

**Keywords:** Tagging, Sei whale, Migration, North Pacific, State-space modeling, Breeding ground, Animal migration, Behavioural ecology

## Abstract

The sei whale (*Balaenoptera borealis*) is an important species among baleen whales in the North Pacific and plays a significant role in the ecosystem. Despite the importance of this species, information regarding its migration patterns and breeding locations remains limited. To enhance the understanding of the phenology of North Pacific sei whales, we deployed satellite-monitored tags on these whales in the western and central North Pacific from 2017 to 2023. We fitted 55 sei whale tracks to a state-space model to describe the whales’ seasonal movements at feeding grounds and their migratory behavior. The whales typically leave their feeding grounds between November and December, with migration pathways extending from off Japan to the west of the Hawaiian Islands. These southward transits converge in the waters of the Marshall Islands and north of Micronesia between 20° N and 7° N, which appear to be breeding grounds. After a brief stay at these breeding grounds, the whales migrate northward from January to February, reaching their feeding grounds around 30°N by March. To the best of our knowledge, this is the first study to present the phenology of feeding and breeding seasons and the migration pattern of North Pacific sei whales.

## Introduction

Migration is a regular and seasonal movement between widely separated and ecologically disparate locations in various animals^1^. Many baleen whales migrate long distances between prey abundant high-latitude feeding areas where phytoplankton bloom and ice-melt in the polar region occur^2–4^ and low-latitude breeding areas by accumulating energy deposit in the blubber^4–10^. Their phenology is highly associated with reproductive and feeding success and with the physical environment. In addition, it is driven by a routine habit that guides seasonal migration^4,8,11–13^. Environmental changes, such as global warming have altered biodiversity and ecosystem^14–18^, and baleen whale behavior depends on prey availability and oceanographic physiology^19–23^. Consequently, understanding the fundamental phenology in baleen whales is necessary to grasp the entire movement strategy. Migration of baleen whales has been well studied in species residing close to the shore during breeding and feeding seasons, such as gray (*Eschrichtius robustus*) and humpback whales (*Megaptera novaeangliae*)^24–29^, however, other offshore species are less-studied and whaling records have sometimes been used^30–32^.

The sei whale (*Balaenoptera borealis*) is a widely distributed oceanic species that can be observed in all subpolar oceans^30,33–36^. Similar to other baleen whales^4,37–40^, this species migrates over long distances between feeding and breeding areas. However, sei whale migration is only reported in North Atlantic sei whales based on relative abundance and satellite-monitored tags, showing the link between the feeding ground in the Labrador Sea and wintering grounds off north-western Africa through Azores Sea^4,36,41^. In the Southern Hemisphere, a study using photographic identification reported that southern sei whales move between the feeding area off Brazil and breeding area in the Falkland Islands. However, much of our knowledge about North Pacific sei whale biology is derived from the whaling period^34^, which provides relevant information about where they were caught**.** Furthermore, our understanding of sei whale phenology is limited compared with other large baleen whales owing to their oceanic distribution.

Current knowledge related to North Pacific sei whale phenology primarily relies on whaling records and discovery marks (also called discovery tags), which were shot into whales and recovered during whaling operations^31,34,42^, proposing the hypothesis that sei whales in the North Pacific arrive at feeding grounds north of 35°N from May to early June. Sei whales spend the summer feeding season in subarctic regions where they feed on copepods, euphausiids, and pelagic fish throughout the entire North Pacific^43–47^. The population size of sei whales is abundant, estimated to be 29,632 individuals in the central and eastern North Pacific from July to August^48^. Therefore, understanding the timing of departure for migration and return to feeding grounds in sei whale phenology is essential for accurately estimating their feeding period.

Sighting survey records of North Pacific sei whales at lower latitudes are limited. They migrate southward from August to early September in the waters west of 160°W. They have also been sighted in the southern waters of Guam, the Northern Mariana Islands, and in the waters of the Hawaiian Islands from fall to winter^49^. Discovery marks also have identified some links of sei whale movements between lower and higher latitudes^31,34^. However, these fragmentary data cannot definitively determine the sei whale breeding grounds, migration pathways, and connectivity between the feeding and breeding grounds. Hence, the winter breeding grounds for sei whales in the North Pacific remain unclear^50^, and further evidence is required to determine their migration patterns.

During the summer feeding season, sei whales are found throughout the Pacific Ocean, from the coast of Japan to the west coast of Canada and the U.S., and the southern part of the Bering Sea, however, they are not present in polar regions and are few in the Bering Sea^31,33,35,51,52^. Genetic analyses using multiple microsatellite DNA loci on the population structure in sei whales from the North Pacific between 145° E and 135° W indicated no separation of the sei whale stock^53,54^. The stock structure of sei whale in the entire North Pacific was also examined based on discovery mark recoveries, distribution and seasonality of catches and oceanographic factors, speculating possible multiple stocks^31,34,55^. Thus, the International Whaling Commission (IWC) hypothesized two cases: single and five stocks including two coastal stocks; however there is no agreement on the stock structure of sei whales in the North Pacific in IWC^55^. To determine the sei whale stock structure, evidence of the connection between the breeding and feeding grounds and latitudinal movement, along with genetic information, is crucial^53,56,57^.

Continuous monitoring of highly-mobile animals is necessary, which is currently only possible through satellite-monitored telemetry^58^. Satellite-monitored tags are a powerful tool, particularly for data-deficient species such as sei whales. Although sei whale tagging has been reported in the Atlantic Ocean^36,41,59^, no reports are available from North Pacific waters, likely because of the challenges of tagging this offshore-distributed species. Herein, we present the first tracking data of North Pacific sei whales throughout the year. Furthermore, we describe the migration patterns, pathways, and possible breeding grounds, along with the latitudinal movements in the feeding area.

## Results

**Figure 1:**
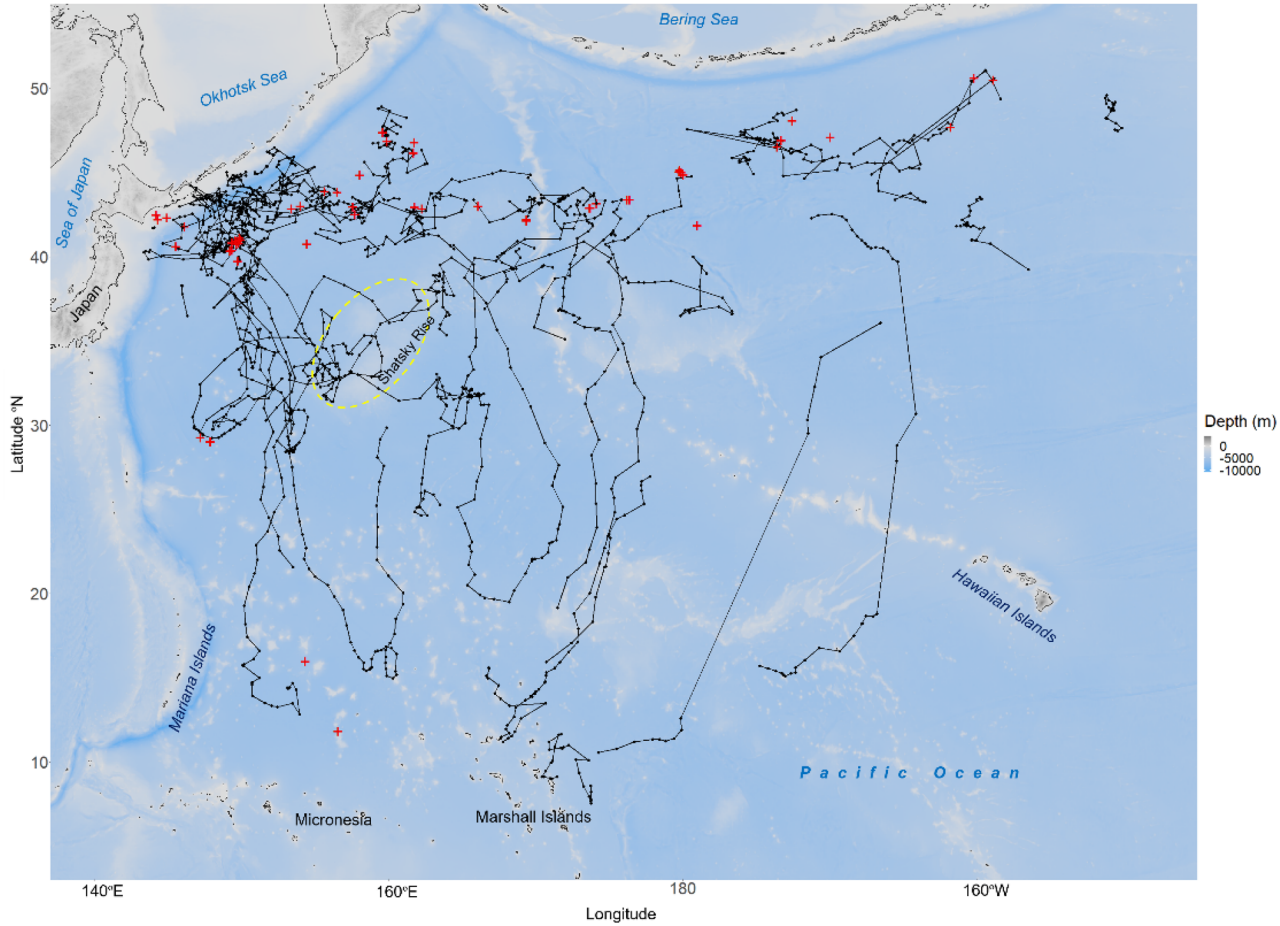
State-space model fits North Pacific sei whale tracks using the correlated random walk model. Red crosses indicate deployment positions, and the tracks cover the distributional area of sei whales in the western and central North Pacific. The fitted locations were mapped and plotted using the marmap package (ver 1.0.1) in R (ver 4.2.2).

Between 2017 and 2022, a total of 55 (16 males, 24 females and 15 unknown) sei whales were tagged in the western and central North Pacific, excluding short tracks lasting < 5 days (Table 1). The duration of these tracks from deployment to last location ranged from 6 to 145 days (male 4–90, female 6–122, and unknown 9–145 days), with a mean (± standard deviation) duration of 45 ± 35.2 (male 41 ± 30.1, female 45 ± 36.3, and unknown 50 ± 37.2). The duration of the ARGOS tracks ranged from 5 to 129 days (male 5–81, female 6–120, and unknown 9–129), with a mean (± standard deviation) duration of 40 ± 33.0 (male 33 ± 28.9, female 40 ± 33.4, and unknown 47 ± 36.9). Several tags initiated the transmissions after a while, assuming that the tag penetrations were deep when the deployments to activate the tags. The sex of these whales was determined through genetic analysis and is also provided in the same table. Figure 1 displays Argos locations fitted to a state-space model with a correlated random walk at a 24-h step, along with their deployment locations. The tracked locations covered a geographical range between 7° N and 50° N latitudes and between 143° E and 150° W longitudes. Notably, no sei whales entered either Bering Sea or Okhotsk Sea, which are separated by the Aleutian Islands and Kuril Islands, respectively. The deployments from different seasons provided a valuable opportunity to investigate the movement of North Pacific sei whales throughout the years, including their feeding and migration periods.

**Table 1:** Data summary for 55 sei whales (*Balaenoptera borealis*) tagged in the western and central North Pacific.

PTT ID	Deployment date	First located date	Last located date	Track duration (days)	ARGOS track duration (days)	Sex
160298	2017-06-25	2017-06-25	2017-08-02	38	38	f
162666	2017-08-26	2017-08-26	2017-10-09	45	44	f
162671	2017-08-28	2017-09-02	2017-10-07	39	34	f
162668	2017-08-30	2017-08-30	2017-09-09	10	10	f
160291	2017-09-07	2017-09-07	2017-09-13	6	6	m
53972	2018-07-04	2018-07-04	2018-07-10	6	6	f
53971	2018-07-05	2018-07-05	2018-08-07	34	33	–
53951	2018-07-08	2018-07-08	2018-07-23	16	15	f
53968	2018-07-25	2018-07-25	2018-08-06	12	12	f
53969	2018-07-25	2018-07-25	2018-08-18	24	24	f
66639	2019-11-07	2019-11-07	2019-11-25	18	18	f
66620	2019-11-09	2019-11-09	2019-11-25	16	16	–
66623	2019-11-10	2019-11-10	2019-12-07	28	28	–
181828	2020-02-25	2020-02-25	2020-04-13	49	48	–
181830	2020-02-25	2020-02-25	2020-06-15	111	111	–
181831	2020-02-25	2020-02-25	2020-03-04	9	9	–
181832	2020-02-25	2020-02-25	2020-03-09	13	13	–
181839	2020-08-05	2020-08-05	2020-08-31	27	27	f
181837	2020-08-11	2020-08-11	2020-08-15	4	4	m
181847	2020-08-21	2020-08-21	2020-09-03	13	13	m
181848	2020-08-22	2020-08-22	2020-09-03	12	12	m
181844	2020-08-27	2020-08-27	2020-11-16	82	81	m
181838	2021-06-19	2021-06-19	2021-07-04	15	15	m
196141	2021-06-19	2021-06-19	2021-07-18	29	29	m
196143	2021-06-22	2021-06-22	2021-07-18	26	25	f
181827	2021-06-22	2021-06-23	2021-08-11	49	49	–
196162	2021-06-23	2021-06-24	2021-07-23	30	30	f
196163	2021-06-24	2021-07-06	2021-09-19	87	75	m
196170	2021-06-25	2021-06-25	2021-09-10	78	78	m
196169	2021-06-25	2021-07-20	2021-08-07	43	18	–
196156	2021-06-25	2021-07-26	2021-08-29	65	34	m
66626	2021-08-25	2021-09-06	2021-10-02	37	26	–
196165	2021-08-27	2021-09-10	2021-10-06	40	25	m
203479	2021-08-29	2021-09-12	2021-09-16	19	5	m
203481	2021-08-29	2021-08-29	2021-09-08	11	11	m
203482	2021-09-10	2021-10-17	2021-11-09	61	23	m
203456	2021-09-12	2021-09-13	2021-10-10	29	28	f
203458	2021-09-13	2021-09-13	2021-10-20	37	37	f
203467	2021-09-13	2021-09-13	2021-10-08	26	26	–
203485	2021-09-13	2021-09-15	2021-10-04	20	18	f
66615	2022-10-20	2022-10-21	2022-11-24	34	34	–
203438	2022-10-21	2022-10-31	2023-01-19	90	80	m
203472	2022-10-22	2022-10-31	2022-11-14	23	15	f
203446	2022-10-23	2022-10-30	2022-11-26	34	27	f
199027	2022-10-23	2022-11-12	2022-11-24	32	12	f
199050	2022-10-23	2022-10-23	2022-12-05	43	43	f
199030	2022-10-24	2022-10-24	2023-02-19	119	118	f
199049	2022-10-26	2022-11-02	2022-12-06	42	34	m
199011	2022-10-29	2022-11-14	2023-03-24	145	129	–
199018	2022-10-30	2022-10-30	2023-01-26	89	88	–
199051	2022-10-30	2022-10-30	2023-01-07	69	69	–
199002	2022-10-30	2022-11-24	2023-02-19	112	87	f
199029	2022-11-07	2022-11-07	2023-02-04	90	90	f
199035	2022-12-10	2022-12-12	2023-04-11	122	120	f
199052	2022-12-12	2023-01-19	2023-04-08	118	79	f

The sex of the deployed animals was determined from biopsy samples. Track duration represents the period from deployment to last locating. ARGOS track duration represents the period of locating whales by ARGOS satellites. Some tags initiated transmission at a later day of deployments. (f = female, m = male, — = unknown).

### Geographical movements

**Figure 2:**
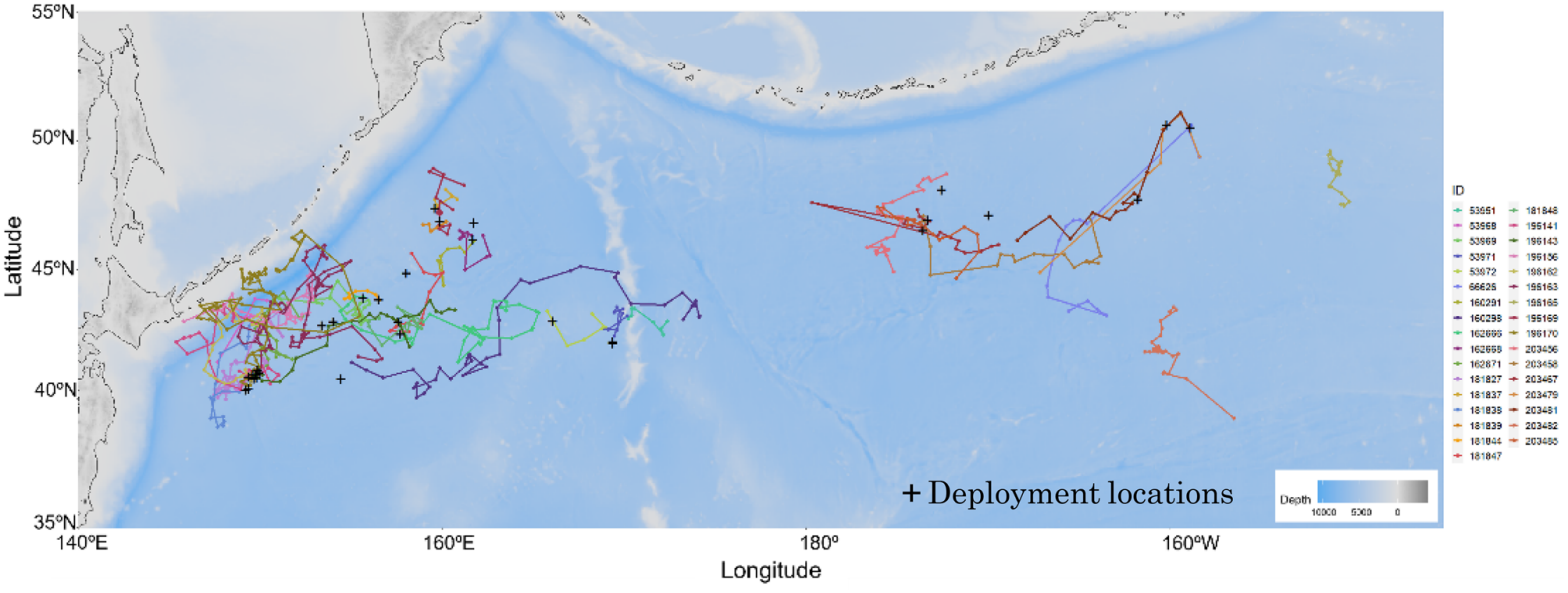
Satellite-monitored tracks of 33 sei whales (*Balaenoptera borealis*) tagged in the western and central North Pacific during the spring to summer seasons of 2017–2022. Deployment locations are marked with black crosses. The fitted locations were mapped and plotted using the marmap package (ver 1.0.1) in R (ver 4.2.2).

**Figure 3:**
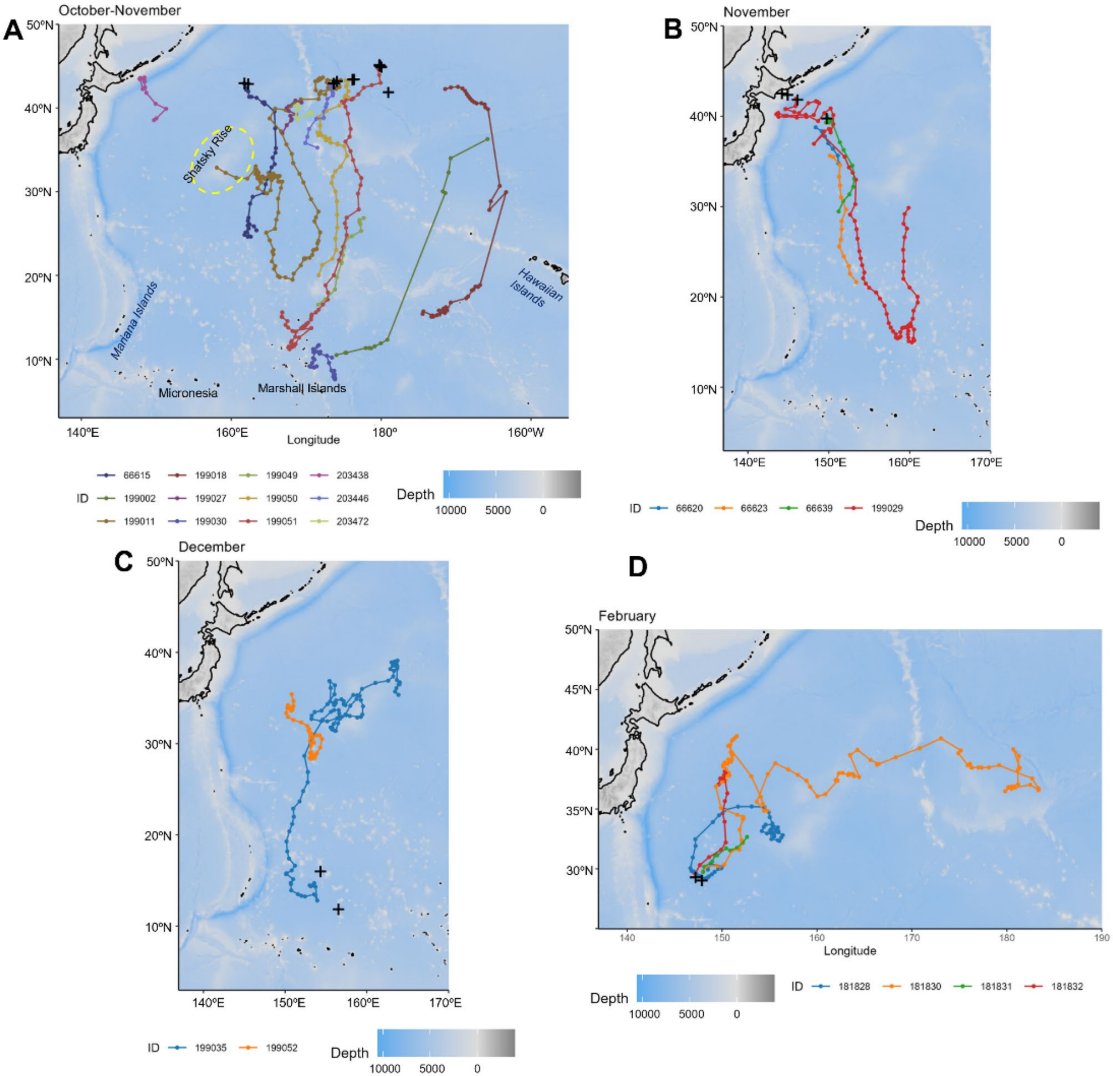
Satellite-monitored tracks of 22 sei whales (*Balaenoptera borealis*) tagged in the western and central North Pacific during the fall to winter seasons of 2017–2022. (**A**) Offshore tagging in October and November. (**B**) Coastal tagging in November. (**C**) Tagging at low latitude in December. (**D**) Tagging in February. Deployment locations are indicated by black crosses. State-space models fitted with correlated random walk were used for mapping. Months and areas are distinguished on the map. The fitted locations were mapped and plotted using the marmap package (ver 1.0.1) in R (ver 4.2.2).

To explore the geographical movement patterns of sei whales, the tracks were divided into two groups based on deployment season: the feeding season, which spans from April to September as shown in Fig. 2, and the migration season, occurring between October and February (Fig. 3). During the spring and summer seasons, sei whales were tagged in the offshore waters of the western and central North Pacific. These tagged whales moved within a range between 35°N and 50°N, and their movements appeared to be non-directional, representing their horizontal movements during the feeding season (Fig. 2).

The tracks from the migration season were divided into four scenes on the maps based on the deployment month and areas to visualize trajectories (Fig. 3). For sei whales tagged in October and November offshore around 160°E and 180°, including two males and three females, most of them initiated a southward migration, and some completed the migration, reaching the southernmost area, likely the breeding area (Fig. 3A). The trajectories spread out in longitude after the deployments, including two east-side trajectories between 180° and 160° W (PTT IDs 199018, 199002). These two whales, including a female (PTT ID 199002), eventually changed the course westward, with one whale reaching the Marshall Islands (Fig. 3A). A tag (PTT ID 203438) began transmitting after the whale moved westward off the coast of northern Japan and remained in the area until the transmission ended in mid-January.

Whales tagged off northern Japan in November also migrated southward, with a female (PTT 199029) completing the southward migration in early January and staying around 15° N. Subsequently, it turned northward until the transmission ended in February, south of Shatsky Rise (Fig. 3B). These southward tracks started off-northern Japan share a migratory corridor band, and the aforementioned female (PTT 199029) used a different pathway during the northward transit.

Two females tagged in December at lower latitudes, south of 20°N, showed northward migration tracks (PTT IDs 199035, 199052), with one track starting after the whale moved far north from the deployment location (PTT ID 199052) (Fig. 3C). These two females eventually reached the waters around the Shatsky Rise and stayed there until the transmissions ended in April.

Four sei whales tagged in February at 30° N all moved northward, with one whale (PTT ID 181830) showing a long transit eastward and crossing 180°longitude, whereas another one (PTT ID 181828) stayed around Shatsky Rise until the transmission ended in April (Fig. 3D).

### Phenology

**Figure 4:**
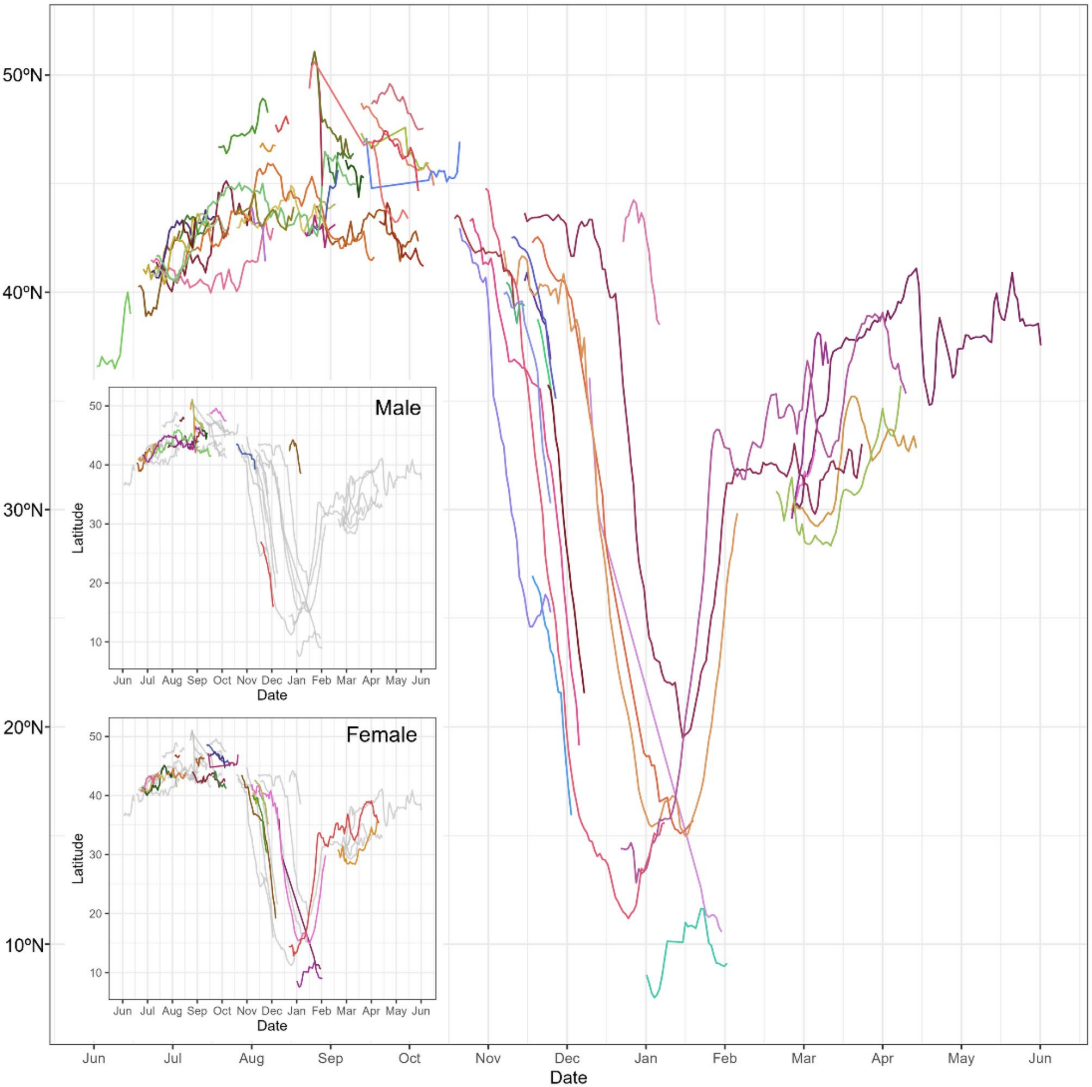
The figure shows latitudinal movements of North Pacific sei whales throughout the year based on satellite monitored tags. Lines were drawn from the fitted data to the state space model. The colored lines in the top sub-panel are showing all known tagged females (n = 24) and their tracks, and further with males and non-sexed animals in grey (n = 31), while the bottom sub-panel with opposite coloration show tracks of all known tagged males (n = 16) in colors, while females and non-sexed animals are in grey (n = 39). From the non-sexed sei whales (n = 15) no biopsy sample was obtained.

**Figure 5:**
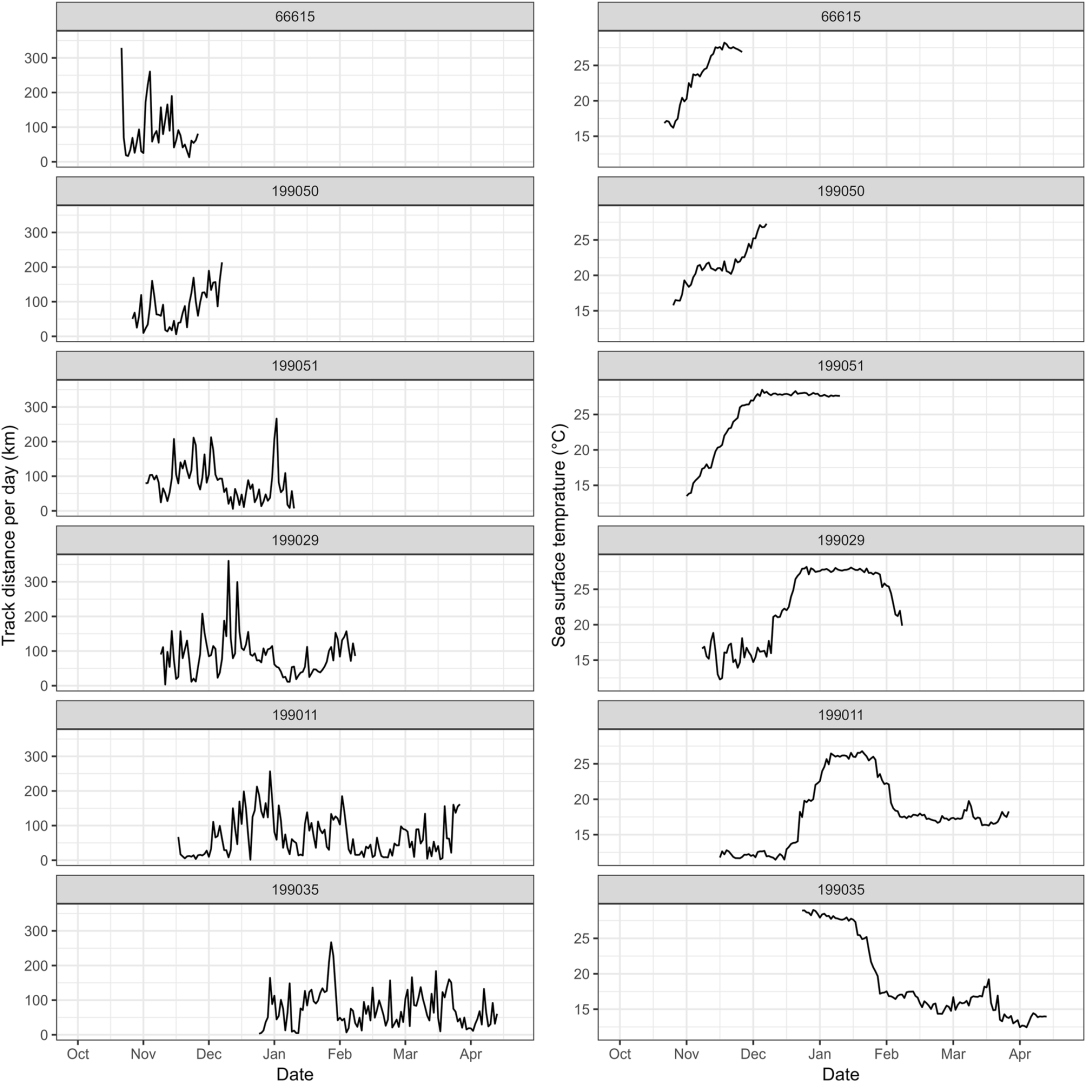
Time series of daily movement distance and sea surface temperature (SST) in six long-tracked sei whales during the winter migration period. Long-tracks are defined as tracks included movements spanning the entire migration route between high-latitude and low-latitude areas.

The phenology of North Pacific sei whales throughout the year has been described, with latitudinal movements plotted for a comprehensive overview (Fig. 4). In addition, a series of travel distances per 24 h and sea surface temperature (SST) at each fitted location were analyzed for six long-tracked whales to identify potential indicators of departure and arrival timings during their migration (Fig. 5). This study focused on sei whale phenology of sei whales, with the correlation between track locations and physical environment are shown in Supplementary Figs. S1 and S2.

The latitudinal movement patterns indicated that sei whales tend to begin their southward migration from late October to December (Figs. 2–5). Some whales reached latitudes lower than 20°N in early December and arrived at the southernmost locations of their trajectories from late December to January. Subsequently, they initiated their northward migration in January, arriving at approximately 30° N, where they ceased northward movements in February when SST ranged between 15 and 17 °C (Fig. 5). Sei whales appear to arrive at their feeding area in February, north of 30°N, and gradually move latitudinally until September, reaching the highest latitudes of 43° N–50° N in August. Subsequently, sei whales gradually shift their distribution southward until October (Fig. 4). This southward shift can be clearly distinguished as part of their migration, as observed through both SST and latitudinal movements (Figs. 4 and 5). Notably, sei whales showing southward migrations are mostly females, whereas short-term tracks are available for males, although a male (TPP ID 199049) also demonstrated a southward transit.

The analysis of the travel distance per day revealed some moving and nonmoving phases for each whale (Fig. 5). However, the daily travel distance was highly varied, making it challenging to pinpoint the exact start and end of migration. In contrast, SST is a useful marker for identifying the transit phase changes. Four whales (PTT IDs 199051, 199029, 199011, and 199035) exhibited SST phase changes that corresponded with their latitudinal movements, with four phases observed as follows: (1) feeding at lower latitudes around 13–15 °C; (2) transit southward with a continuous temperature rise to around 27 °C; (3) breeding at high SST maintained for a month; and (4) transit northward with continuous SST decreasing to around 17 °C (Fig. 5).

**Figure 6:**
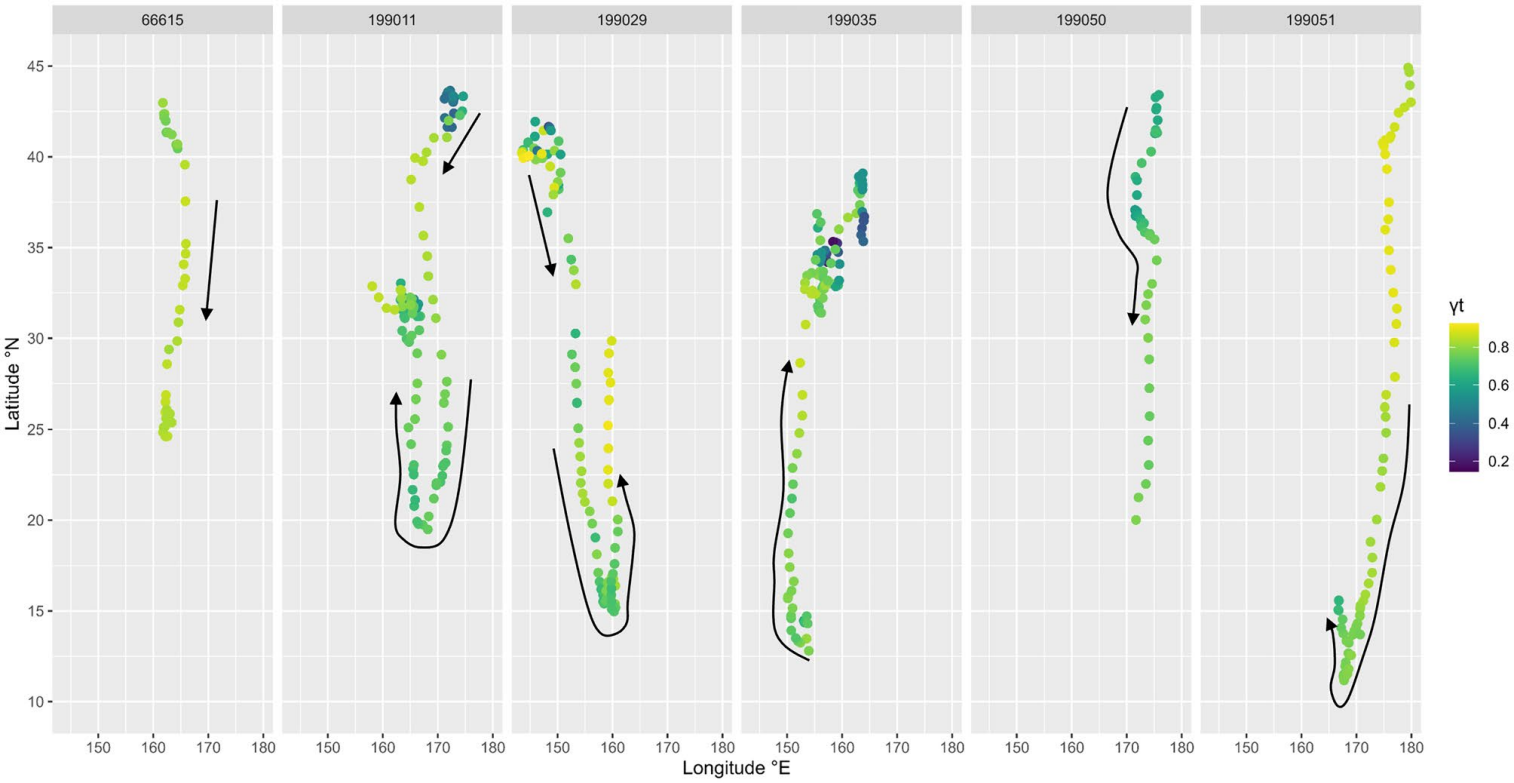
Migration patterns and switching state-space model with movement persistence γ_t_ (ranging from 0 to 1) in six long-tracked sei whales included movements spanning the entire migration route between high-latitude and low-latitude areas. Arrows indicate the direction of trajectories.

The migration of sei whales is also indicated by their latitudinal movements with behavioral persistence *ɣt* (Fig. 6). A searching behavior with low *ɣt* was observed in two whales before initiating southward transits (PTT IDs 199011, 199029). The migration distances, which were determined by the initial migration to the southernmost locations, were approximately 2480 and 3850 km, respectively. During transit, unidirectional and fast movement behavior was observed, whereas the searching behavior was observed in the feeding area at higher latitudes after the northward travel. The value of behavioral persistence in the breeding area appears to fall between that of the feeding and transition phases, indicating that the movements of sei whales at the breeding grounds differ from those at the feeding grounds. The migration behaviors of these whales were similar, i.e., they moved quickly during transit and slowed down in the breeding area (Fig. 6). The duration of stay in the breeding area, which is generally lower than 20°N, ranged from approximately half a month to 2 months (Fig. 4).

### Breeding area

**Figure 7:**
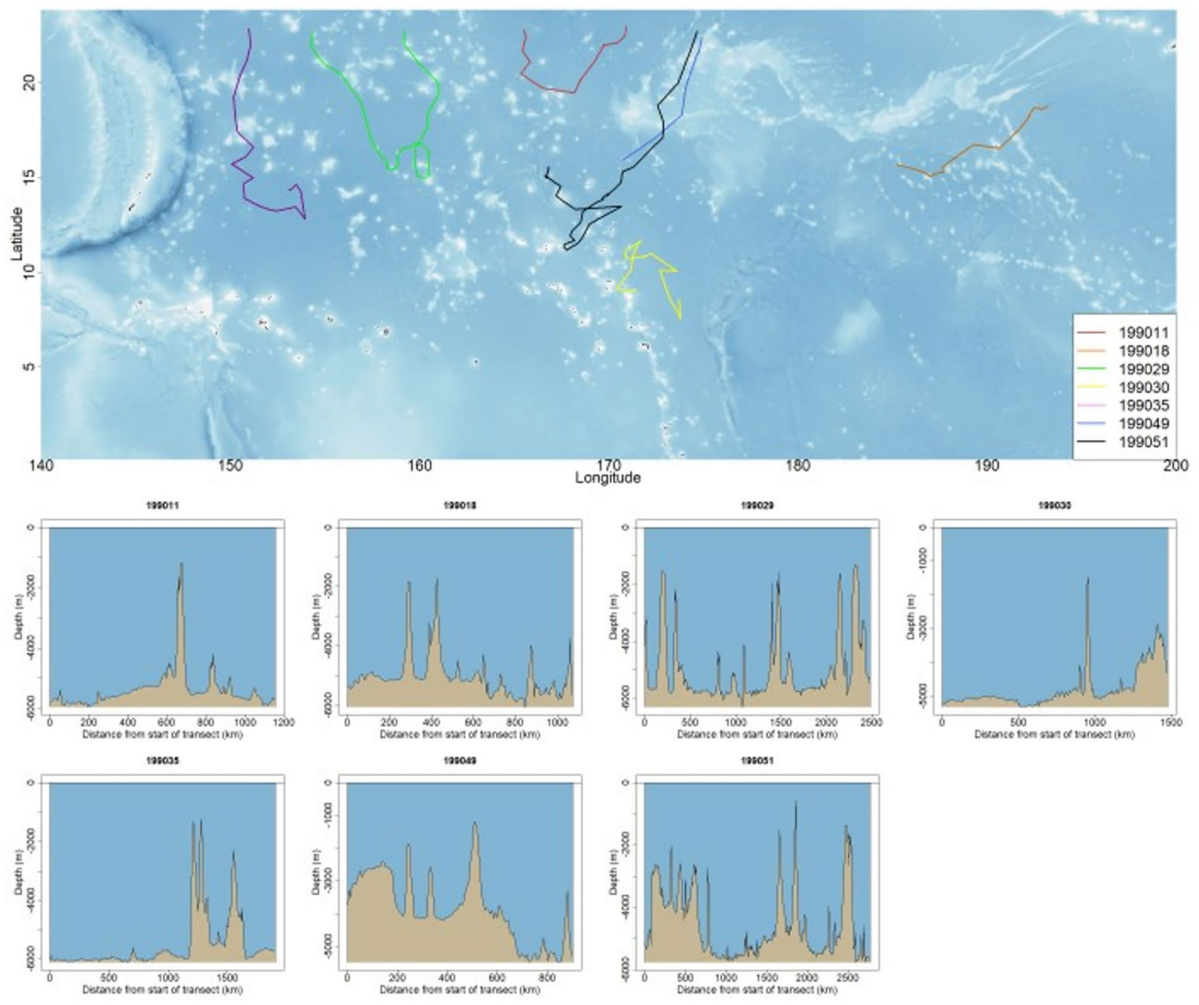
Tracks of seven sei whales at latitudes lower than 23° N (upper panel) and topographic features along the route of each track (lower). The fitted locations were mapped and plotted using the marmap package (ver 1.0.1) in R (ver 4.2.2).

The tracks of sei whales indicated that their presumed breeding area was located the southernmost area on their trajectories, which they do not reach but head in the same direction. This area includes regions around the Marshall Islands and the north of Micronesia between 7° N and 20° N, and it appears to be an important breeding ground, with the Marshall Islands being particularly prominent. This large area encompasses many atolls, with SST being higher than 25 °C (Figs. 3, 5, and 7). To examine the topographic features along the sei whale tracks in the breeding area, the water depth at each location along the tracks was analyzed (Fig. 7). The closest distances from the track locations of two sei whales (PTT IDs 199051, 199030) to atolls in the Marshall Islands are approximately 5 and 15 km, respectively. This analysis revealed that sei whales tended to occupy areas with topography deeper than 1000 m.

## Discussion

This study provided the first comprehensive description of annual movement patterns in western-central North Pacific sei whales, highlighting their basic phenology, breeding areas, and previously unidentified migration pathways^33,50^. The waters around the Marshall Islands and north of the Federated States of Micronesia serve as breeding grounds for western-central North Pacific sei whales, as some tracks showed arrivals and returns from these southernmost areas. During the feeding season, sei whales are typically distributed between 30° N and 50° N, south of the Aleutian Islands and east of Japan and the Kuril Islands, consistent with previous reports based on sightings and discovery mark surveys^34,52,60,61^. Our data further confirmed that sei whales in the North Pacific are strongly associated with the North Pacific Basin, with their northern distribution limited to the south of the Aleutian Islands. This northern limit is relatively lower than that of Atlantic sei whales, which is around 60°N off Greenland^59^.

The latitudinal movements of sei whales during the feeding season were revealed in our data. They appeared to arrive at the southern part of the feeding area. Subsequently, they shift their distribution to the north and later to the south until they initiate their migration at the end of October. Although this movement pattern is known to be influenced by prey availability and environmental factors^60,61^, specific distribution factors during the feeding season warrant further investigation using track-based analysis.

The intensive deployments conducted in the fall of the 2022 season in our study provided valuable insights into the migration pathways of western and central North Pacific sei whales to their breeding grounds. These migrating pathways are distributed over a broad longitudinal range exceeding 20° in longitude, indicating that sei whales do not follow major, well-defined migratory pathways. However, sei whales exhibited widespread southward pathways that eventually converge at the breeding area. Further investigation is necessary to gain a comprehensive understanding of their migration behavior. Because only a small number of sei whales have been sighted in November around Hawaiian waters^49,62^. These sightings likely correspond to sei whales on their southward migratory pathways during the fall-to-winter season, as indicated by our tracking data. This wide range of pathways contrasts with the pathway of the Western North Pacific gray whale, which uses a fairly constricted migratory route between the feeding ground off Sakhalin Island in the Okhotsk Sea and the breeding ground off Baja California^63^. In our data, most tracks during breeding migration belonged to females, whereas male tracks were fragmentary, making it challenging to determine the male migration pattern. However, a male track (TPP ID 199049) indicated that they also migrate southward during the same period. In this study, comparison of migration patterns between males and females was difficult owing to the small sample size. Thus, further investigation is necessary to comprehensively understand sei whale migration and reproductive behaviors.

Sei whales arrived between 20° N and 7° N for about 1 month. During their time in the southernmost breeding area, sei whales tended to stay in waters deeper than 1000 m, with surface water temperatures around 27 °C for a relatively short period before changing their course northward. Despite the presence of numerous atolls in the Marshall Islands, whales do not venture into shallow waters around these atolls. Previous sightings of sei whales in the waters of Guam and the Northern Mariana Islands south of 20°N indicated that the recognized sei whale breeding grounds in our study could extend to the waters around the Mariana Islands, as they are located at similar latitudes^64^. These findings indicated that sei whales feeding in the western and central North Pacific have a broader breeding range at lower latitudes, unlike humpback whales, which concentrate in smaller areas during their breeding season^65–67^. A mother with a calf was sighted in the south of 30°N from February to April^34^, supporting that the southern migrating tracks are their possible breeding area. In contrast to the presumed sei whale breeding ground in our study, humpback whale breeding grounds are shallower < 200 m^68–71^. These findings indicate that sei whales do not need shallow waters for breeding.

The annual tracks allowed us to determine the feeding period based on migration timings of departure and arrival at the feeding grounds and how they shift their feeding areas during the feeding season. The favorable temperature for sei whales in the feeding area is approximately 13–15 °C^35^. Based on the behavioral states of the tracked sei whales and their horizontal movements, they presumably begin to forage after arriving in waters around 30°N near Shatsky Rise. Although further tagging study is needed to determine when and how they initiate feeding during or after migration, the timing of departure and arrival to the feeding areas, the feeding period is assumed to be 8 months, which is longer than previously reported^34^. These different interpretations likely stem from the lack of discovery mark data during the migration periods of February to April and November to January, as well as the difficulty in distinguishing latitudinal movement in the feeding ground and transit movement to breeding grounds from sightings and discovery mark data^34^. Adding to the fundamental phenology, migration, feeding and reproductive behaviors are highly involved^72,73^. For understanding the responses of sei whales to climate change and their role in the ecosystem in the North Pacific, the phenology with specific feeding behavior is becoming more important.

One remaining question in sei whale phenology concerns the conception period and its location. Previous research indicated that the mean conception and birth times are from late December to the beginning of November^34,52^. Applying this period to our results suggests that conception might occur on the way to the breeding grounds, with births taking place at the breeding grounds. Humpback whales give birth at the breeding grounds^74^. From whaling records at north of 20° N, female sei whales exhibit variations in the timing of departure based on their sexual maturity stages, including mature, pregnant, resting, and lactating stages in the lower latitude, and a mother with a calf was observed in February to April, suggesting a nursing period^34^. Although, individual sexual maturity data are not available in this study, whether sei whales display spatiotemporal variations in their migration patterns among different sexual maturity stages remains to be determined.

Masaki^34^ proposed stock separation by longitudes based on different migration patterns among the waters west of 180°, 180°–160° W, and east of 160° W. However, our data revealed a long eastward movement of a sei whale tagged in February, suggesting that some whales undergo large-scale longitudinal movements within the feeding area. This implies that the stock structure may require more sophisticated genetic analysis for conclusive determination, and additional tracking data in the eastern North Pacific will contribute to a better understanding of the entire North Pacific sei whale stock structure.

Our results contribute to the understanding of sei whale biology, particularly their life cycle, feeding, and reproductive status. The migration phenology of sei whales is also useful for planning future surveys, considering the selection of seasons and areas. In addition, further tracking of sei whales in the eastern North Pacific will enhance our understanding of their movement across the entire North Pacific, as more than one migration pattern by latitudinal areas has been reported^34^. Intensive tagging during winter and spring at offshore areas on northward migration in sei whales might track dispersal to feeding grounds to determine how they spread throughout the North Pacific feeding grounds. Whale migration patterns are determined by their reproductive status, sexual maturity, prey distribution, and energy accumulated in the feeding area^4,6,8,34,75,76^. These variations related to migration should be examined for a better understanding of sei whale phenology. Environmental changes and food availability are of great concern for migrating baleen whales^8,12,13,29,77^, and long-term monitoring of sei whale migration patterns through tagging experiments is useful for investigating the impact of environmental change.

## Methods

### Tag deployment

Tag deployments occurred between spring and fall during the feeding season at higher latitudes and in winter at lower latitudes across a wide expanse of the western and central North Pacific from 2017 to 2022 (Table 1; Fig. 1). We used implantable satellite-monitored tags encased in stainless steel housing with a triangular stop plate (SPOT177, 98 mm × 22 mm, Wildlife Computers, Redmond, Washington, USA) for tagging sei whales. Only this type of tag provides long-time movements for monitoring migration patterns and deployment seasons were divided to cover seasonal movement throughout years. To secure the tags during implantation, anchors were designed and affixed to the front of the tag housing. The tags were programmed to transmit data at a rate of 250 transmissions per day, with either 45 or 30 s between repetitions. The tags were deployed using the ARTSC carrier in conjunction with the whale tag launcher ARTS^78^ and were released from a height of 6.5 m on the bow deck of the vessels^78^. All experiences were conducted by well-trained survey researchers and crews to minimize disturbance and maximize success. Skin biopsies were simultaneously obtained after the deployment of the satellite tags using a biopsy Larsen gun system^79^. These samples were subsequently used for molecular sexing and other chemical analyses. Biopsy samples collected outside Japan’s EEZ were transported to Japan following the necessary procedures under CITES. The sex of the deployed animals was determined using the method outlined by Abe et al.^80^, with a slight modification that incorporates the microsatellite locus GT23^81^ instead of GATA417 as a negative control^82^. All fieldwork experiments were conducted in compliance with permissions granted by the Fishery Agency of Japan (Suikan: 19-601, 30-371, 2-604, 2-2144, 2-2143, 3-176, 3-262, 3-2527, 3-3198, 3-3199, 4-1030, 4-2225-2, 4-2729) and the original survey plan of Research Plan for New Scientific Whale Research Program in the western North Pacific (NEWREP-NP) was reported to the IWC and reviewed by the panel (SC/67A/REP/01 available on IWC web page). The tagging for individual migration in the context of stock structure hypotheses was recommended by the panel. Tagging was performed in accordance with the guidelines proposed by IWC^83^. All experimental protocol, including platforms and tag deployments, was approved by the Fishery Agency of Japan and the Institute of Cetacean Research. This study is reported in accordance with ARRIVE guidelines.

### Argos data processing and analysis

We collected location data from the Argos satellite system, which included location quality classes 3, 2, 1, 0, A, B, and Z. We excluded whales that had been tracked for less than 5 days and locations that were deemed inconceivable. The Argos locations, location quality, and measurement errors were fitted to a state-space model (SSM) to account for the uncertainty of Argos-based locations and to estimate unobservable behavior states at fixed time intervals. This was performed using the R package *aniMotum* ver.1.1^84,85^. Initially, we used the *fit_ssm* function to fit the data to a continuous-time correlated random walk (crw) SSM and estimate the locations at 24-h intervals with a speed threshold (vmax) of 4 ms^−1^ to filter all the locations. We then routed these locations using the *route_path* function to correct on-land locations based on geographical data. If a convergence problem occurred, we adjusted the intervals to 25-h or 23 h. We also used the *fit_ssm* function to fit a move persistence (mp) model to calculate move persistence *γ*_t_ (ranging from 0 to 1)^84^ for successful long-tracks that included movements spanning the entire migration route between high-latitude and low-latitude areas. This helped us identify transitory and resident movement behaviors. Finally, we calculated the distance between the estimated locations from the fitted locations by SSM using the package *argosfilter* ver.0.7^86^.

The fitted locations were mapped and plotted using the marmap package (ver 1.0.1), and the time-series locations were plotted alongside environmental data obtained from the ERDDAP server using the rerddapXtracto package 1.1.4^87^. SST data were sourced from the JPL MUR MEaSUREs Project 2015. GHRSST Level 4 MUR Global Foundation SST Analysis. Ver. 4.1. PO.DAAC, CA, USA. Dataset accessed [2023-04] at https://doi.org/10.5067/GHGMR-4FJ04^88^. Topographic data were obtained from the ETOPO 1 Arc-Minute Global Relief Model^89^. All calculations were conducted in R (ver. 4.2.2) and with the tidyverse package tidyverse ver 2.0.0.^90^ were also used for data handling and plotting.

### Supplementary Information

Supplementary Figures.

## Data Availability

The data used in this study are available from the corresponding author upon reasonable request.
