# Author Correction: Annual phenology and migration routes to breeding grounds in western-central North Pacific sei whales

**DOI:** 10.1038/s41598-024-64744-8

**Published:** 2024-06-17

**Authors:** Kenji Konishi, Shingo Minamikawa, Lars Kleivane, Megumi Takahashi

**Affiliations:** 1 The Institute of Cetacean Research, 4-5, Toyomi-cho, Chuo-ku, Tokyo, 104-0055 Japan; 2 Fisheries Resources Institute, Japan Fisheries Research and Education Agency, 2-12-4, Fukuura, Kanazawa-ku, Yokohama-shi, Kanagawa 236-8648 Japan; 3 LKARTS-Norway, Skutvik Landhandel, 8290 Skutvik, Norway

Correction to: *Scientific Reports* 10.1038/s41598-024-61831-8, published online 16 May 2024

The original version of this Article contained an error in the name of Megumi Takahashi, which was incorrectly given as Megumi Takahahsi.

The original Article has been corrected.

