# Supplementary material for: Annual phenology and migration routes to breeding grounds in western-central North Pacific sei whales
Source: Sci Rep. 2024 May 16;14:11212. doi: 10.1038/s41598-024-61831-8 (PMC11098811; doi:10.1038/s41598-024-61831-8)
Supplement: Supplementary file 1 — Supplementary Figures. [file 41598_2024_61831_MOESM1_ESM.docx]

Relationship between the tracked locations in sei whales and environmental factors

To explore the relationship between sei whale tracks and physical environment, the correlation matrices are shown in two migrating (November – January) and non-migrating (February – October) periods determined by the latitudinal movement pattern of tracked sei whales in Fig. 4. The location data fitted to a state-space model (SSM) in this study are used.


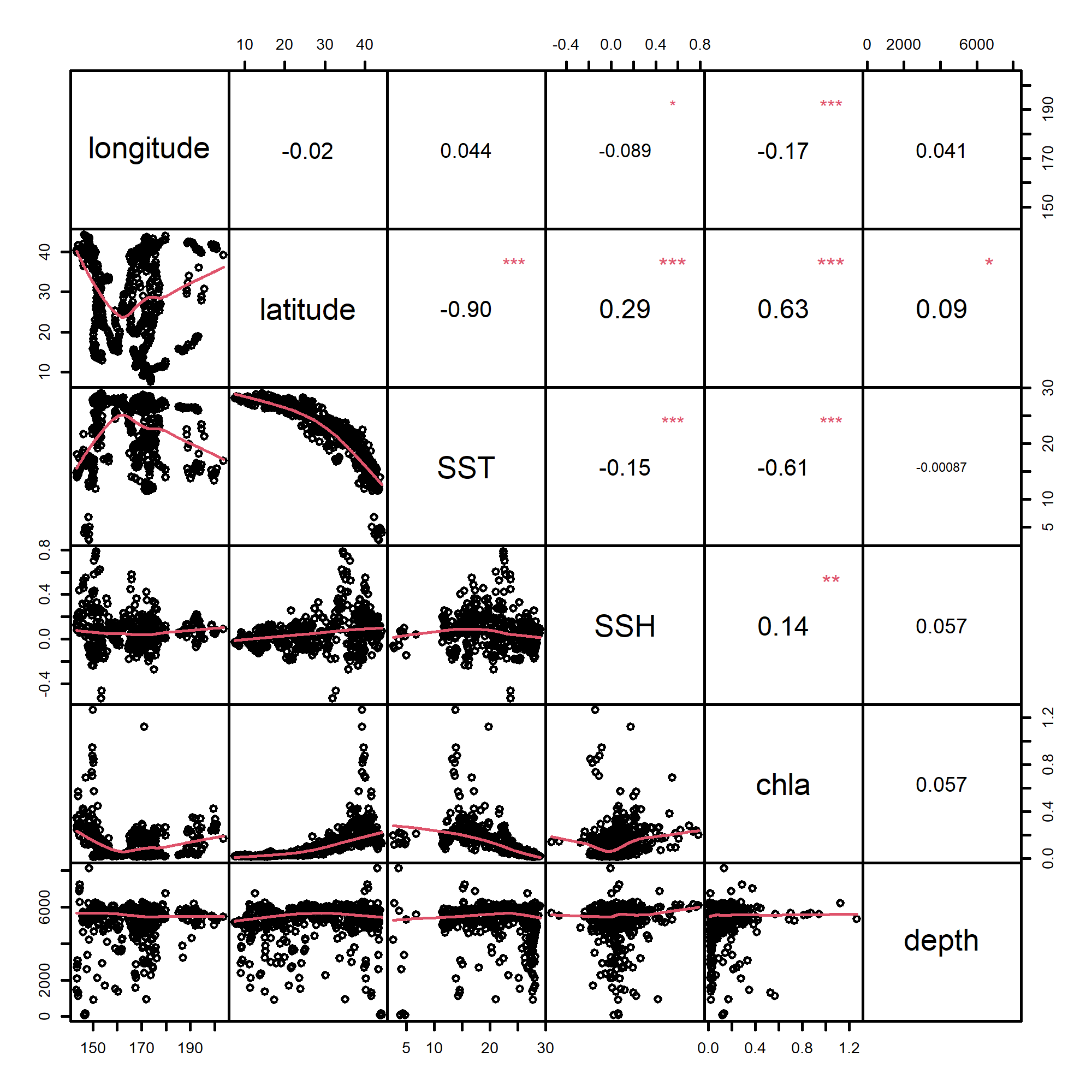


Fig. S1. Correlation matrix for tracked locations and physical environment during the migrating period from November to January.


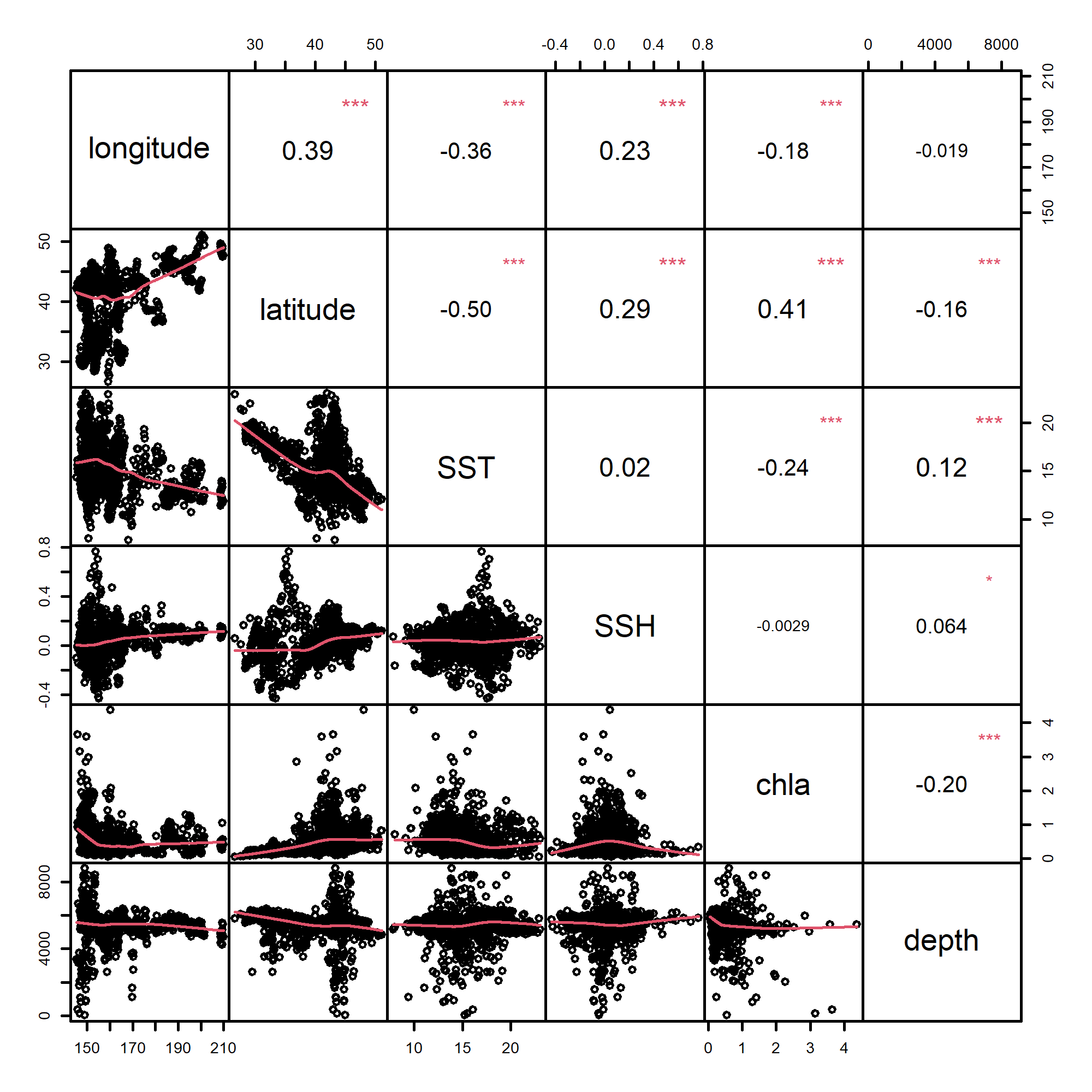


Fig. S2. Correlation matrix for tracked locations and physical environment during the non-migrating period from February to October.

Fig. S1. Summary of physiological variables used in this Supplementary.
